# Draft Genome Sequence of Bacillus halotolerans IcBac2.1, a Strain with Potential as a Phytopathogen Control Agent

**DOI:** 10.1128/mra.00857-22

**Published:** 2022-10-31

**Authors:** Miriam Memenza-Zegarra, Ernesto Ormeño-Orrillo, Doris Zúñiga-Dávila

**Affiliations:** a Laboratorio de Ecología Microbiana y Biotecnología, Departamento de Biología, Facultad de Ciencias, Universidad Nacional Agraria La Molina, Lima, Peru; SIPBS, University of Strathclyde

## Abstract

Several species of the genus *Bacillus* are used as plant growth-promoting bacteria. In particular, species of the subtilis group are known as good antagonists of phytopathogenic fungi. Here, we report the draft genome sequence of a rhizospheric *Bacillus* strain with promising abilities as a biocontrol agent.

## ANNOUNCEMENT

*Bacillus* strain IcBac2.1 was isolated from common bean (Phaseolus vulgaris) rhizosphere soil collected in the coastal desert of Ica, Peru (13°36′01″S, 76°01′34″W) (1). IcBac2.1 has *in vitro* activity against Rhizoctonia solani, Fusarium oxysporum, and Sclerotinia sclerotiorum (2), reduces the incidence of the latter under field conditions (1), and produces inhibitory nonvolatile amphiphilic compounds (3). To improve the taxonomy of IcBac2.1 using genome similarity metrics and to explore its gene repertoire for antifungal compound biosynthesis, we decided to sequence its genome.

IcBac2.1 (= B02) was obtained in 2016 by thermal treatment of a soil suspension and plating of serial dilutions on tryptone-glucose-yeast-extract (TGE) agar (1, 2). It has since been preserved in 25% glycerol at −80°C in our laboratory. A culture was grown from the original stock in tryptic soy broth at 28°C and 150 rpm and checked for purity by observing cell morphology and Gram stain reaction. DNA was obtained with the GeneJET genomic DNA purification kit (Thermo Fisher Scientific, USA) following the manufacturer’s instructions. A library prepared with the Nextera DNA Flex library preparation kit (Illumina, USA) and constructed without DNA shearing or size selection was sequenced in a MiSeq instrument, which yielded 420,392 paired-end reads with an average length of 270 bp. Reads were quality trimmed with Trimmomatic v0.4 (4). An assembly was generated with SPAdes v3.15.3 (5), evaluated with QUAST v5.2 (6) and BUSCO v5.2.2 using the bacillales_odb10 data set (7), and annotated with the NCBI Prokaryotic Genome Annotation Pipeline (PGAP) v6.1 (8). Digital DNA-DNA hybridization (dDDH) and BLAST-based average nucleotide identity (ANIb) values were calculated using the Genome-to-Genome Distance Calculator (GGDC) v3.0 (9) and JSpeciesWS v3.9.6 (10), respectively. A phylogenomic tree was constructed with PhyloPhlAn v3.0 (11). Secondary metabolite biosynthetic gene clusters (BGCs) were predicted with antiSMASH v6.1.1 (12). Antibiotic resistance genes and pathogenicity factors were evaluated with PathogenFinder v1.1 (13). Unless otherwise stated, default parameters were used for bioinformatic tools.

**FIG 1:**
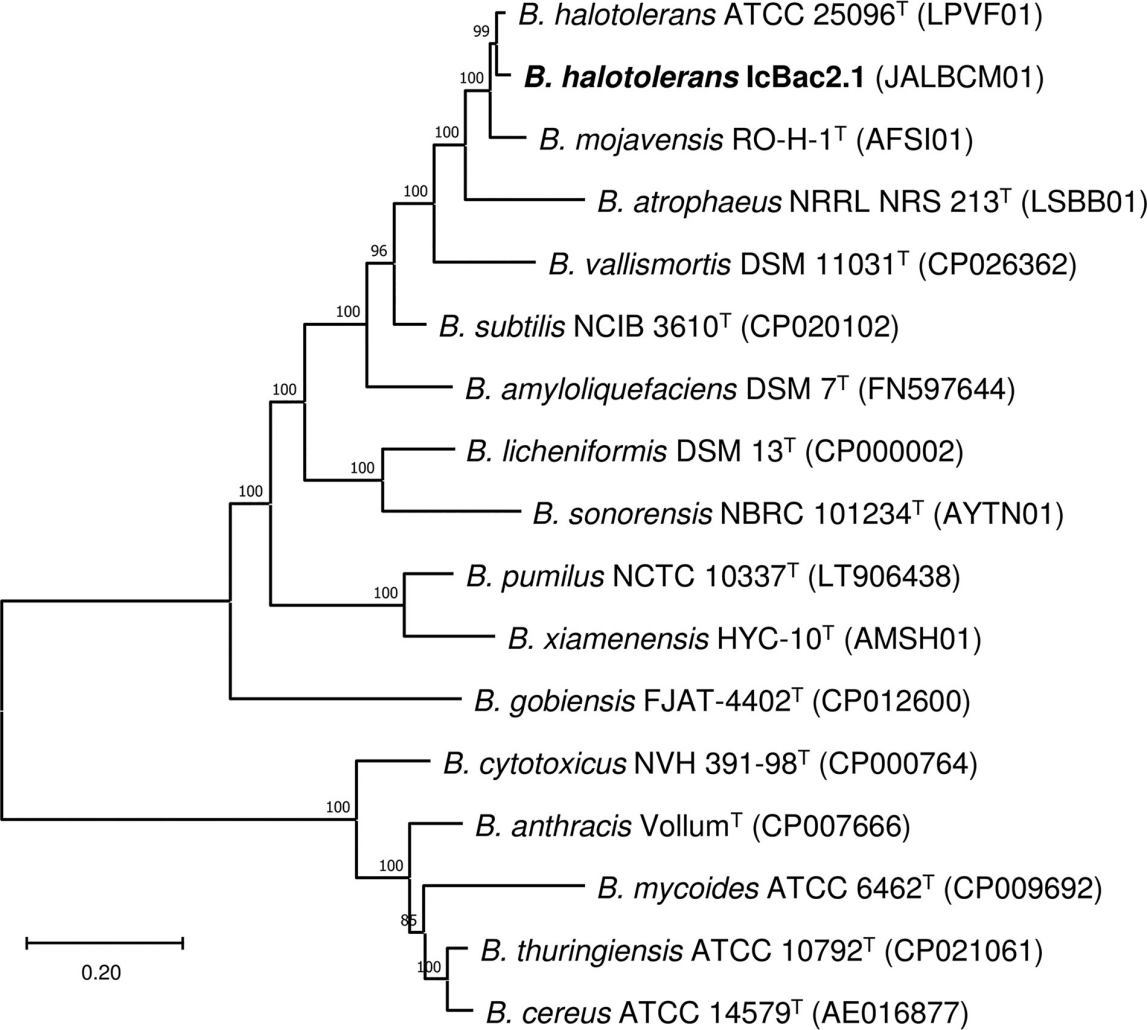
Phylogenomic tree showing the relationships between strain IcBac2.1 and type strains of *Bacillus* species. The phylogeny was constructed with PhyloPhlAn v3.0 using the supermatrix pipeline, phylophlan database, and medium diversity options. Branch labels correspond to Shimodaira-Hasegawa-like support values.

The 4,048,647-bp genome assembly was distributed in 64 contigs, with a *N*_50_ value of 180,125 bp, a GC content of 43.76%, and 30× coverage. It contained 4,070 coding sequences (CDSs), 75 tRNA genes, and 18 rRNA genes. Genome completeness was estimated with BUSCO at 99.8%. A phylogenomic tree showed that IcBac2.1 is more closely related to Bacillus halotolerans than to Bacillus mojavensis (Fig. 1). Its sequence showed 90.2% dDDH and 98.52% ANIb with respect to that of B. halotolerans ATCC 25096^T^ (GenBank accession number LPVF00000000), values that indicate that IcBac2.1 belongs to this species.

Nineteen BGCs for secondary metabolites, encompassing 12.3% of the genome, were found. Four had 100% similarity to known BGCs for the siderophore bacillibactin and the antimicrobials subtilosin A, bacilysin, and bacillaene. Other clusters showed similarity of >61% to BGCs for surfactin and the antifungals fengycin and pliplastin. Seven clusters were not similar to known BGCs but contained genes related to polyketide or terpene synthesis. The PathogenFinder program predicted IcBac2.1 as a nonhuman pathogen.

The genome sequence of IcBac2.1 will aid in the rational utilization of this promising bacterium, as well as providing useful information on the biogeography and ecology of B. halotolerans, because it represents both the first from a Southern Hemisphere strain and the first from a P. vulgaris rhizosphere isolate.

### Data availability.

The assembled genome sequence of B. halotolerans IcBac2.1 was deposited in GenBank under accession number JAMXBU000000000. Raw sequence reads were submitted to the NCBI SRA under accession number SRX15687688. Project data are available under BioProject accession number PRJNA848436 and BioSample accession number SAMN29005956.
